# Enhancing Spam Message Classification and Detection Using Transformer-Based Embedding and Ensemble Learning

**DOI:** 10.3390/s23083861

**Published:** 2023-04-10

**Authors:** Abdallah Ghourabi, Manar Alohaly

**Affiliations:** 1 Department of Computer Science, Jouf University, Sakaka 72388, Saudi Arabia; aghourabi@ju.edu.sa; 2 Higher School of Sciences and Technology of Hammam Sousse, University of Sousse, Sousse 4011, Tunisia; 3 Department of Information Systems, College of Computer and Information Sciences, Princess Nourah bint Abdulrahman University, P.O. Box 84428, Riyadh 11671, Saudi Arabia

**Keywords:** SMS classification, spam detection, Transformer, Ensemble Learning, GPT

## Abstract

Over the last decade, the Short Message Service (SMS) has become a primary communication channel. Nevertheless, its popularity has also given rise to the so-called SMS spam. These messages, i.e., spam, are annoying and potentially malicious by exposing SMS users to credential theft and data loss. To mitigate this persistent threat, we propose a new model for SMS spam detection based on pre-trained Transformers and Ensemble Learning. The proposed model uses a text embedding technique that builds on the recent advancements of the GPT-3 Transformer. This technique provides a high-quality representation that can improve detection results. In addition, we used an Ensemble Learning method where four machine learning models were grouped into one model that performed significantly better than its separate constituent parts. The experimental evaluation of the model was performed using the SMS Spam Collection Dataset. The obtained results showed a state-of-the-art performance that exceeded all previous works with an accuracy that reached 99.91%.

## 1. Introduction

Nowadays, more and more people are using text messages (SMS) as a means of communication and marketing. According to a recent article published by Slicktext [1], five billion people around the world use SMS, which is about 65% of the entire human population. This number is estimated to reach 5.9 billion users by 2025. The proliferation of SMS has coincided with a rise in malicious activities such as spam and SMiShing. In addition to being an annoyance to users, these activities can have serious financial consequences for individuals and businesses [2]. Generally, the senders of these spam messages aim to obtain personal or financial information via SMS that may include fraudulent content, a malicious link, or malware. According to statistics published in February 2023 [3], the volume of spam has significantly increased through the decade. There were 1.27 million spam messages sent in September 2021. In comparison, in August 2022, 10.89 billion spam messages were sent. Regarding financial losses, an estimated USD 10,066,331,169 was lost in 2021 due to spam texts.

Several research works have been proposed in recent years to address the detection of SMS spam. Various techniques have been used in these works, which start from classical filtering methods (such as blacklisting, whitelisting, or heuristics) to advanced artificial intelligence algorithms. Although traditional filtering techniques are simpler and faster, artificial-intelligence-based techniques are more effective in terms of detection rate, especially when dealing with renewed spam content. The idea is to create intelligent models based on machine learning algorithms that can analyze the content of messages and classify them as ham or spam. The performance of the proposed models varies depending on several factors, including the text representation technique used, the feature selection method, the type of classification algorithm, etc. The right choice of these criteria is very crucial to achieving good classification results. Our goal is to develop a robust model capable of achieving state-of-the-art results in spam detection. In this context, we studied several word embedding and classification methods. Finally, we decided to use a text embedding technique based on a Transformer model and a classification method employing the Ensemble Learning strategy.

Recently, Natural Language Processing (NLP) has evolved greatly due to the development of pre-trained language models (known also as Transformer models) such as BERT and GPT. One of the most significant turning points in the history of natural language processing was the release of BERT [4] in 2018 by Google. As a result, researchers accomplished top results and provided high-performance models in various tasks, including question answering, text classification, sentiment analysis, and machine translation. BERT opened the door for the appearance of other types of Transformers such as the famous GPT and its three generations that followed. The third generation GPT-3 [5] was released in 2020 with the aim of providing human-like text. The advantage of GPT-3 is its enormous number of learning parameters and the large amounts of data it has been trained with. Our idea is to use one of the Transformer models to encode text messages into high quality vector representations before starting the classification process. For this reason, we tested both BERT and GPT-3 models. The results were very satisfactory for both, with a slight superiority in favor of GPT-3. On the other hand, we tried to design a classification model that was as robust as possible. The idea that interested us the most was using an Ensemble Learning strategy. This involves using several classifiers together and gathering their output results to decide on the final output according to a specific voting technique.

In this paper, we propose a new model capable of improving the results of SMS classification and spam detection. We first employed a recent text embedding technique based on the GPT-3 Transformer to represent the text message in a dense numerical vector. Then, we gathered four classifiers (SVM, KNN, CNN and LightGBM) in an Ensemble module to classify the vector representations obtained from the previous module. To make the right decision regarding the input instance, we created a weighted voting algorithm that collected the results of the four classifiers and calculated the most suitable output.

The following is a summary of the main contributions of our work:

- The proposal of an advanced model that can improve the detection rate of spam messages.
- The use of a recent technique for text embedding based on a pre-trained language model that is able to recognize the similarity between words in the message and provide a high-quality numerical representation.
- The development of an Ensemble Learning strategy using an optimized weighted voting technique to help achieve better classification results.
- The proposed model outperformed previous works and achieved a remarkable accuracy of 99.91%.

The rest of the paper is structured as follows: Section 2 covers the related works. Section 3 explains some background details. The architecture of our model is outlined in Section 4. The outcomes of the experiments are discussed in Section 5. Finally, Section 6 is dedicated to the conclusion.

## 2. Related Work

In recent years, several research works have been proposed in the field of SMS spam detection and classification. In these works, several machine learning techniques were used that involved Naive Bayes [6,7,8], deep learning [9,10], the Hidden Markov model [11], recent pre-trained language models [12,13], etc. In this section, we try to briefly describe the main works carried out on this research topic.

In previous works [9,12], we proposed models to detect spam messages written in Arabic and English. The first approach consisted of stacking Convolutional Neural layers with a Long Short-Term Memory layer in order to create a hybrid deep learning classifier; the second approach consisted of utilizing the pretrained language model “BERT” in association with a MLP network to classify collected messages. The experimental evaluation showed an accuracy of 98.37% with the first model and 99.45% with the second model.

In [10], deep learning algorithms were used by Roy et al. to determine which SMS messages should be marked as spam and which should be ignored. They combined the Convolutional Neural Network (CNN) and the Long Short-Term Memory (LSTM) deep learning methods. The objective was to establish a system for distinguishing between spam and legitimate messages sent via SMS. Some machine learning algorithms such as Gradient Boosting, Naive Bayes, Random Forest, Stochastic Gradient Descent, and Logistic Regression were used to compare them with their method. According to the findings, the CNN and LSTM models outperformed other machine learning models by a wide margin.

The authors of [6] proposed a model that they called “Smishing Detector” to detect SMiShing messages with the aim of minimizing false positives. There were four distinct parts to the proposed model. The first component was devoted to scanning incoming text messages for malicious content and keywords using the algorithm of classification known as “Naive Bayes”. The second component was for analyzing the messages’ URLs. The third one was for delving into the message-related website’s source code. The final component was a download detector that checked if there was a malicious APK associated with a download link. The authors tested the model empirically and found it to be 96.29 percent accurate.

Another model for identifying SMiShing messages was proposed by Joo et al. [7]. There are four parts to this model: a module for keeping track of the activities via short message service, an analyzer to examine the content of the messages, a determinant for classifying and blocking SMiShing text messages, and a database for storing all that information. The algorithm of classification used in this model was Naive Bayes.

In [2], the authors presented a model to detect SMiShing messages using machine learning algorithms; they called it “SmiDCA”. The authors of this model opted to utilize correlation algorithms to select the 39 most important features from SMiShing messages. As a next step, they used four machine learning classifiers to assess how well their model performed. The experimental results of the Random Forest classifier showed a 96.4% accuracy.

To improve the performance of text message classification methods, the authors of [14] suggested a processing method for normalizing and expanding textual messages. This procedure was founded on semantic analysis, semantic dictionaries, and disambiguation methods. The primary goal was to extend the original text by standardizing the words and creating new attributes while minimizing potential performance-detrimental factors, such as redundancies and inconsistent wording.

In [8], Naive Bayes and FP-growth were used by Arifin et al. to propose a strategy to filter out SMS spam. The FP-growth technique is intended to extract the frequent itemset from the text messages, while the Naive Bayes algorithm is employed to categorize messages and filter out the spam messages. The experimental evaluation of this method demonstrated an overall accuracy of 98.5%.

In [11], the authors suggested a Hidden Markov model with a weighting feature mechanism to filter spam messages. Their approach was to create a word weighting algorithm that took into account the dissimilarities in word distribution between ham and spam. Based on this algorithm, an SMS word labeling technique was then employed to generate an HMM observation sequence. According to the experimental findings, this model achieved an accuracy equal to 96.9%.

In [13], Liu et al. relied on the Vanilla Transformer to propose a modified version of this model to detect SMS spam. With this model, they used the GloVe method to create vector representations for text messages. Experiments conducted with this model showed an accuracy of 98.92%.

Table 1 provides a brief overview of the various works presented. Despite the importance of these works, the design of our new model and the choice of techniques used have allowed us to achieve better performance. Indeed, our approach stands out for taking advantage of Transformer-based models to provide high-quality text representation, which has not been well explored in previous works. Moreover, the combination of multiple classification algorithms together favors increasing the number of true positives and decreasing the number of false negatives. To the best of our knowledge, the approach presented in the current article is the first to combine Transformer-based text embedding with Ensemble Learning for spam detection.

## 3. Background

### 3.1. Generative Pre-Trained Transformer (GPT)

The GPT [5,17,18] is a pre-trained language model based on a Transformer architecture developed by OpenAI. The model is pre-trained on a large corpus in order to perform different NLP tasks. The GPT architecture contains a 12-layer, decoder-only transformer with a masked, multi-headed self-attention module. The multi-headed self-attention mechanism is applied on the input context tokens in association with position-wise feedforward layers to generate an output distribution over target tokens. The first generation of the GPT [17] was released in 2018. It was trained on a BooksCorpus dataset containing about 7000 unique unpublished books, and it was fine-tuned for various tasks such as text classification, question answering, natural language inference, and semantic similarity. In 2019, OpenAI released the second generation of the GPT (GPT-2) [18], which was an extension of its predecessor with more learnable parameters. GPT-2 had 1.5 billion parameters (10 times more than the first GPT) and was trained on a high volume of web data that totaled 40 GB in size. This enabled it to achieve top results for various language modeling tasks. The great success of the GPT was especially noticed with the release of its third generation (GPT-3) in 2020 [5]. The GPT-3 is based on the same model and architecture as the GPT-2 and features a few modifications. The number of learnable parameters has become larger, reaching up to 175 billion parameters. Due to the enormous number of parameters, the GPT-3 has been able to be trained with huge amounts of text from the Internet on a wide range of topics and subjects [19]. As a result, the GPT-3 has been able to learn different subjects of documents and generate articles that are difficult for human reviewers to distinguish from articles written by humans.

**Figure 1:**
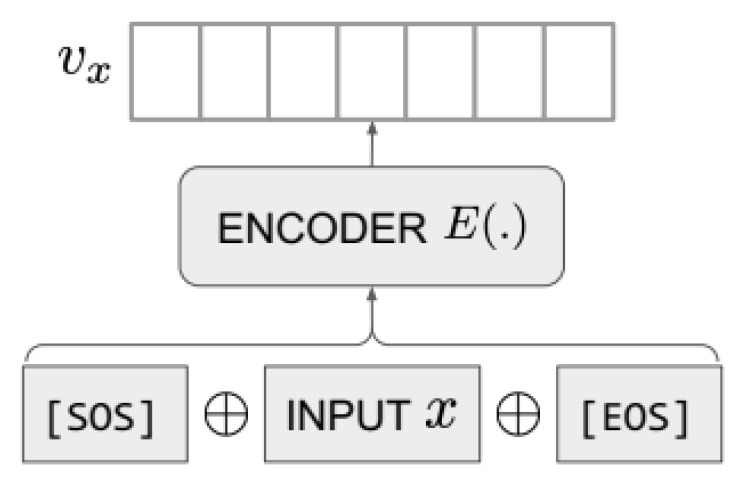
Text Representation with the embedding model of GPT-3 [20].

Recently, in January 2022, OpenAI released a paper describing its new model for text embedding [20]. This model, based on the GPT-3, aims to provide high quality vector representations of text and code. The advantage of the embedding technique used is that it is able to capture the semantic similarity of pieces of text. Figure 1 describes the overall embedding process of this model. Consider an input text *x*. Two special tokens are added to *x*, which include [SOS] at the beginning and [EOS] at the end of the input text. Then, a Transform Encoder *E* maps the input sequence to a dense vector representation $V_{x}$. The embedding of the input text is determined by extracting the hidden state from the last layer that corresponds to the special token [EOS]. Experimental results demonstrated that this model produced new state-of-the-art results in linear-probe classification.

The great success of the GPT-3 and its embedding model encouraged us to adopt it in our approach to represent input messages in order to achieve a higher classification accuracy.

### 3.2. Ensemble Learning

Ensemble Learning is a learning algorithm that trains multiple learners to predict a solution for the same problem [21]. An ensemble model includes a number of learners considered as weak learners, which are generally called base learners. The added-value of ensemble learning is in its ability to combine several learners to form a high-performance learner whose accuracy is superior to its constituent parts. The creation of the base learners is generally done by training the data with learning algorithms such as Neural Network, Naive Bayes, Decision Tree, SVM, or other machine learning techniques. Some ensemble methods employ one learning algorithm to form homogeneous learners. Other methods employ different learning algorithms to form heterogeneous base learners [21]. The last method is especially useful if we want to take advantage of the strengths of each learning algorithm. To achieve good performance with ensemble learning, base learners generally need to be as accuracate and diverse as possible.

Typically, an ensemble architecture includes multiple base learners that each take an instance vector *x* as an input and a method for aggregating or combining their results to produce a single output prediction *y*. In the literature, there are several methods of combination and aggregation. In the following, we introduce four of the most-used techniques: Boosting, Bagging, Stacking, and Voting.

#### 3.2.1. Boosting

Boosting is an algorithm family that includes several variants, of which AdaBoost is the most known in this category. This algorithm starts by giving each of the training samples equal weights and then corrects them over several rounds. Let $D_{t}$ be the weight distribution during the *t*-th learning round. The algorithm uses these weights to create a base learner $h_{t}$, which it then tests with training samples to increase the weights of the mis-classified samples. In this way, a new weight distribution $D_{t+1}$ is created. The algorithm then generates another base learner from $D_{t+1}$ by invoking the base learning algorithm again. This process is repeated *T* times, and the final learner is obtained using weighted majority voting among the *T* base learners [21]. A summary of the boosting procedure is explained in Algorithm 1.   
**Algorithm 1:** Boosting algorithm.
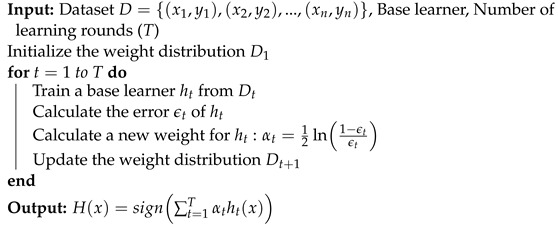


#### 3.2.2. Bagging

The Bagging technique consists of using a learning algorithm to train a number of base learners, which each derive from a different training set called the “bootstrap sample”. This sample is derived by involving uniform substitution with replacement from the initial data set. As a result, for a bootstrap sample, certain training examples might show up, but others might not, and there is a probability of roughly 0.632 that an example will appear at least once. After generating the base learners, the bagging algorithm combines them by using the majority voting technique and predicts the class that received the most votes [21]. Algorithm 2 summarizes the process of the Bagging method.   
**Algorithm 2:** Bagging algorithm.
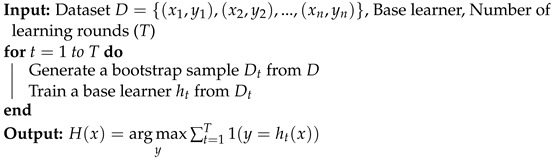


#### 3.2.3. Stacking

Stacking is another ensemble learning method in which a number of individual base learners, called “first-level learners”, are trained from the training dataset by using various learning methods. Then, a meta-learner known as a “second-level learner” is used to combine these individual learners [21]. Algorithm 3 describes the process of this ensemble technique.   
**Algorithm 3:** Stacking algorithm.
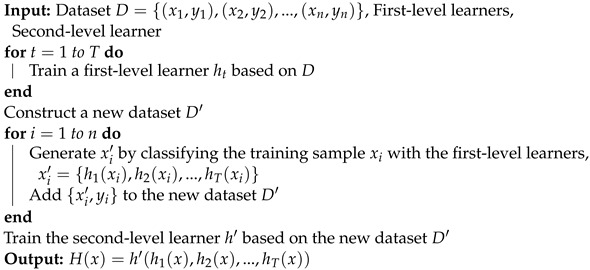


#### 3.2.4. Voting

Voting is a common method of combining in ensemble learning. For explanations, consider the example of a classification problem with *x* as the input vector and *c* as the class label. Assume that we have a set of *T* individual classifiers $\left\{h_{1},\cdots ,h_{T}\right\}$, and our goal is to combine these classifiers to predict the final output class from a set of possible classes of size *l*$\left\{c_{1},\cdots ,c_{l}\right\}$. Suppose as well that the output of each learner $h_{i}$ is a vector of dimension *l* equal to $\left(h_{i}^{1}\left(x\right),\cdots ,h_{i}^{l}\left(x\right)\right)$, where $h_{i}^{j}\left(x\right)$ is the output value related to the class $c_{j}$ by applying the classifier $h_{i}$ to the input instance *x* [22]. The objective of our method is to combine the results of all the classifiers to predict the most appropriate class through a voting process. In the following, we describe three popular voting techniques: Majority Voting, Weighted Voting, and Soft Voting.

- Majority Voting:

In Majority Voting, each classifier casts a vote to elect one class, and the class that receives more than half of the votes will be considered the final output class. If none of the classes gets more than 50% of the votes, the classification operation will be rejected. The formula for selecting the final output H(x) of the ensemble classifier in Majority Voting is described in the following equation [22].

(1) $$H\left(x\right)=\left\{\begin{array}{cc} c_{j} & \mathrm{if}\sum_{i=1}^{T}h_{i}^{j}\left(x\right)>\frac{1}{2}\sum_{k=1}^{l}\sum_{i=1}^{T}h_{i}^{k}\left(x\right)\\ \mathrm{rejection} & \mathrm{otherwise} \end{array}\right.$$

Alternatively, we can determine the final class by looking for the class that receives the highest number of votes instead of half the votes. This method is called Plurality Voting:

(2) $$H\left(x\right)=c_{\underset{j}{\arg\max}\sum_{i=1}^{T}h_{i}^{j}\left(x\right)}$$

- Weighted Voting:

In the case where the classifiers perform differently, it is more judicious to give more importance to the classifiers that have better performance. This may be accomplished by a weighted vote. The output result can be calculated as follows [22]:

(3) $$H\left(x\right)=c_{\underset{j}{\arg\max}\sum_{i=1}^{T}w_{i}h_{i}^{j}\left(x\right)}$$

where $w_{i}$ is the weight given to each classifier $h_{i}$.

- Soft Voting:

Soft voting can be used in the case where the classifiers have probabilistic or scoring outputs. In this method, the output prediction is obtained by calculating the average of the output results of all the classifiers in the ensemble. For each class $c_{j}$, the final score is calculated as follows:

(4) $$H^{j}\left(x\right)=\frac{1}{T}\sum_{i=1}^{T}h_{i}^{j}\left(x\right)$$

## 4. Proposed Model

**Figure 2:**
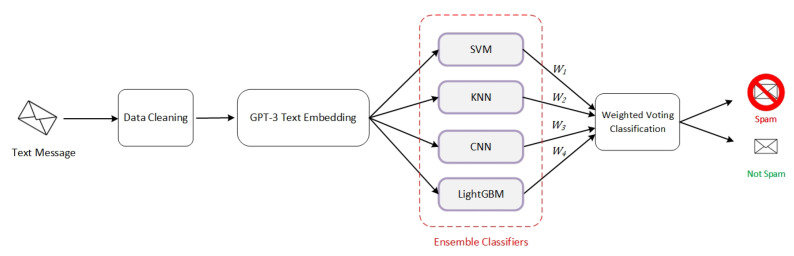
Architecture of the spam detection model.

In this section, we describe in detail the model we propose in the current article. The objective of this model is to classify short messages through two main phases: the conversion of textual messages into dense numerical representations and the use of an ensemble model that combines several machine learning algorithms to classify these representations and identify spam messages with high accuracy. Figure 2 and Algorithm 4 illustrate the design and the functioning principles of the proposed model. We first cleaned the text messages and convert them into numeric formats using an Embedding operation based on a pre-trained Transformer. Then, an ensemble learning method containing four different classifiers was applied to the embedding output for classification purposes. The final decision regarding the class of the input message was determined with a Weighted Voting technique applied to the results of the four classifiers.
**Algorithm 4:** Message embedding and classification.
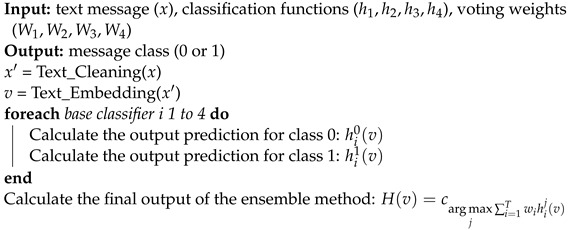


### 4.1. Text Cleaning

Text cleaning is an important step before proceeding with data processing. It consists of removing unnecessary elements from text messages. Items to be removed include punctuation marks, unnecessary symbols, trailing whitespaces, line breaks, etc. This operation is useful to improve the understanding of text by the machine learning models.

### 4.2. GPT-3 Text Embedding

Text embedding is an encoded representation of a text, which consists of transforming the textual content into a vector of numbers in a meaningful way. This operation is essential if we want to analyze textual data with machine learning algorithms. In the field of Natural Language Processing (NLP), several text representation techniques are well known, including TF-IDF, word embedding models such as Word2Vec [23], GloVe [24], and fastText [25], or the more recent methods based on pre-trained Transformer models such as BERT [4] and GPT [5]. Since our approach requires the use of a text embedding method, we investigated and compared these different techniques. We found that the methods based on Transformers, especially the third generation of the GPT, performed better than the other models in terms of classification results. Furthermore, in a recent paper published in January 2022, the creators of the GPT-3 showed that their text embedding method led to high quality vector representations of text and achieved new state-of-the-art results in linear-probe classification over the best previous text embedding models. This encouraged us to integrate this method in our solution.

Consider a text message *x* and an embedding function noted *E*; the output of the embedding process is obtained as follows:

(5) v=E(x)

where *v* is a numerical vector of size 1536 representing the mapping of the message *x* through the embedding function *E*.

The encoder *E* is a Transformer model based on the GPT-3 that is trained on paired text data where semantic similarity and contextual relevance were considered. In this way, the model can capture the semantic similarity of pieces of text, which is very useful in text classification and clustering tasks.

### 4.3. Ensemble Classifiers

The idea of assembling several classifiers is very efficient to obtain a performing and stable model. It can take advantage of the strengths of each classifier to make the best decision regarding the input instance. In the case of our approach, we chose to use an ensemble method containing four base classifiers for spam message detection. The selection of these four classifiers was performed after studying different machine learning algorithms, including Decision Tree, SVM, Logistic Regression, K-Nearest Neighbors (KNN), AdaBoost, Extra Trees, Random Forest, a gradient boosting algorithm (LightGBM), and deep learning algorithms (CNN and LSTM). The selection was based mainly on the accuracy results. Finally, four classifiers were chosen to be part of the ensemble strategy: SVM, KNN, LightGBM, and CNN.

- Support Vector Machines (SVM) [26] are a set of supervised learning techniques for solving classification and regression problems. They have become very popular due to their ability to produce good accuracy results and to handle high dimensional data. The goal of SVM is to find the best hyperplane in an N-dimensional space that accurately classifies the data points.
- K-Nearest Neighbors (KNN) [27] is a non-parametric supervised learning technique applied to classification and regression problems. KNN is one of the simplest machine learning algorithms. It consists of classifying the input into the category that is most similar among the available categories. The decision regarding the chosen class is based on the selection of the k-nearest neighbor of the input object.
- The Light Gradient Boosting Machine (LightGBM) [28] is an open-source distributed gradient boosting framework that was developed by Microsoft in 2017. It operates using decision trees and may be applied to a variety of machine learning problems, including regression, classification, and ranking. LightGBM is distinguished by its ability to handle large datasets while guaranteeing fast training speeds and low memory usage.
- The Convolutional Neural Network (CNN) [29] is a type of deep learning architecture known for the use of a mathematical operation called convolution in its layers. It is commonly used for image classification and recognition tasks, but it can also be used for NLP tasks. The CNN architecture consists of several layers, including convolutional layers, pooling layers, and fully connected layers. The advantage of the convolutional layer is its ability to extract relevant features from data.

### 4.4. Weighted Voting Classification

As described in the previous section, four classifiers were employed together to predict the class of the input message. The results of these classifiers were not necessarily the same. To combine the outputs of the classifiers, there are several methods. In our model, we opted for the Weighted Voting technique to make the most suitable decision regarding the input message. The Weighted Voting method consists of assigning various weights to the base classifiers according to specific criteria and taking a vote of the classifiers’ outputs with the consideration of their weights. Usually, the highest weight is assigned to the best performing classifier.

To explain the Weighted Voting process, consider *x* as an input instance for each of the four classifiers, $h_{1},h_{2},h_{3}$, and $h_{4}$ as classification functions associated respectively with the SVM, KNN, CNN and LightGBM classifiers, and $W_{1},W_{2},W_{3}$, and $W_{4}$ as weights assigned to each of the four base classifiers. The final output H(x) of the ensemble method is determined as follows:

(6) $$H\left(x\right)=c_{\underset{j}{\arg\max}\sum_{i=1}^{4}w_{i}h_{i}^{j}\left(x\right)}$$

The obtained result provides the final decision about the class associated with the input message: $C_{0}$ (not spam) or $C_{1}$ (spam). This decision must take into account the output and the weight of each base classifier.

The challenge with this ensemble method lies in how to find the combination of weights that results in better performance than any other combination. Several techniques have been proposed to solve this problem, such as using the formula $W_{i}=\frac{Accuracy_{i}}{\sum_{j=1}^{T}Accuracy_{j}}$ to determine the ratio of the accuracy of each base classifier to the total sum of all the accuracies, or applying the method of the Grid Search to find the optimal weights among a grid of different values. In this work, we used Bayesian optimization to find the best weights for our ensemble model.

Bayesian optimization is a sound optimization technique [30]. In our previous work [31], we tested this technique, and the results were very satisfactory. In the case of our model, the optimization problem is characterized as:

(7) $$x^{*}=\underset{x\in \chi }{\arg\max}f\left(x\right)$$

where $x^{*}$ identifies the weights of the ensemble model that need to be optimized. The search space of the weights is indicated by the symbol χ. The objective function f(x) represents how well the ensemble model performs given the chosen weights. Accuracy is the evaluation criterion used to gauge how well the objective function performs. Therefore, the goal of the optimization is to identify the collection of weights $x^{*}$ that will enable the function f(x) to obtain the best performance.

## 5. Experimental Evaluation

To evaluate the model proposed in the current paper, we present in this section the results of the experiments performed. These experiments contained various machine learning algorithms that were tested with four types of text representation: TF-IDF, Word embedding, BERT-based embedding, and GPT-3-based embedding.

### 5.1. Dataset Description

The different experiments presented here were conducted with the SMS Spam Collection Dataset [15]. This collection contains a total of 5574 SMS messages, of which 4827 are legitimate and 747 are spam messages. This dataset was collected by Almeida et al. [15], and it has been publicly available to help researchers test their solutions for spam messages. Currently, it is the most-used dataset in research works interested in the classification and detection of spam messages.

### 5.2. Evaluation Measures

To evaluate our solution, we employed four common classification metrics: accuracy, precision, recall, and the F1-score. We also included a confusion matrix to present the distribution of the classification results.

- *Accuracy*: the number of true positives and true negatives (correct predictions) divided by the total of predictions.
  (8) $$Accuracy=\frac{\left(TruePositives+TrueNegatives\right)}{\left(TruePositives+FalsePositives+FalseNegatives+TrueNegatives\right)}$$
- *Precision*: the proportion of true positives among the total positive predictions.
  (9) $$Precision=\frac{TruePositives}{TruePositives+FalsePositives}$$
- *Recall*: the percentage of positives instances.
  (10) $$Recall=\frac{TruePositives}{TruePositives+FalseNegatives}$$
- *F*1-*Score*: a harmonic mean combining precision and recall in a single measure.
  (11) $$F1-Score=\frac{2\times Precision\times Recall}{Precision+Recall}$$
- The *confusion matrix* can be described as a table determining the number of true and false predictions.

### 5.3. Results

To evaluate the performance of our solution and compare it with other models, we conducted several experiments involving different types of text representation and machine learning algorithms. During this experimental evaluation, ten classification algorithms were tested: Support Vector Machine, K-Nearest Neighbors, Decision Tree, Logistic Regression, Random Forest, AdaBoost, Bagging, LightGBM, CNN, and LSTM. Each of these classifiers was tested with three types of text representation: (i) TF-IDF and Word embedding for deep learning algorithms, (ii) embedding based on the BERT transformer, and (iii) embedding based on the GPT-3 transformer. The objective was to compare the performance of the classifiers with each embedding method and determine which one was the most efficient. In all experiments, the same distribution of data was used: 80% for training and 20% for testing. Four evaluation measures were calculated to compare the classifiers: Accuracy, Precision, Recall, and the F1-score.

Table 2 shows the results obtained from the experimental tests. By interpreting these results, we can notice that the embedding methods based on the transformers BERT and especially GPT-3 had considerably improved the Accuracy of all the classifiers. For example, for the SVM algorithm, the Accuracy increased from 0.978475 with TF-IDF to 0.993722 with BERT embedding and 0.996413 with GPT-3 embedding. Similarly, the Precision, Recall, and F1-score respecitvely reached 1.000000, 0.972973 and 0.986301 with GPT-3 Embedding. Concerning the LightGBM classifier, the Accuracy was improved by 2% by switching from TF-IDF to GPT-3 embedding; the Precision, the Recall, and the F1-score obtained their maximum values as well with this embedding. The same improvements were noticed with the two deep learning algorithms CNN and LSTM. With Word embedding, they obtained an Accuracy of 0.986547 and 0.987444, respectively; with BERT embedding, the Accuracy increased to 0.993722 and 0.991031, respectively; they reached their maximum with GPT-3 embedding at obtained values of 0.997309 and 0.993722 for the CNN and LSTM, respectively.

**Figure 3:**
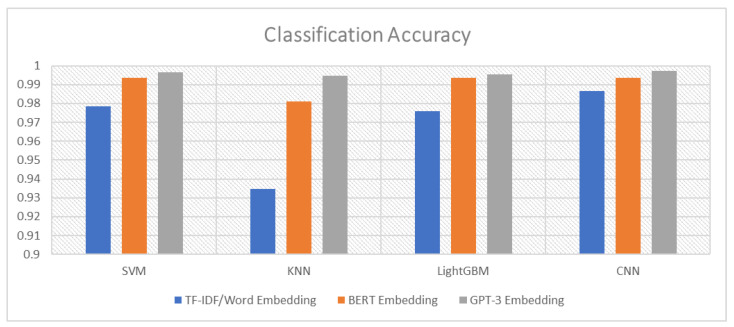
Accuracy of each classifier with the 3 types of embedding.

**Figure 4:**
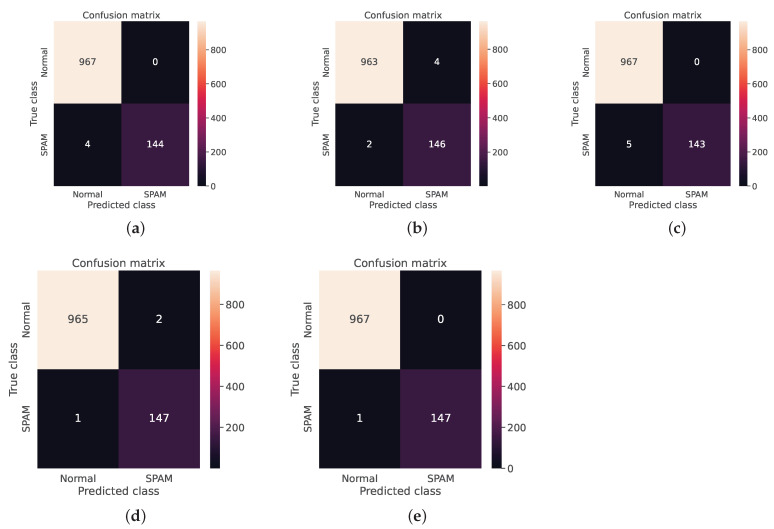
Confusion matrix. (**a**) Confusion matrix of SVM; (**b**) confusion matrix of KNN; (**c**) confusion matrix of Light-GBM; (**d**) confusion matrix of CNN; (**e**) confusion matrix of voting ensemble.

As mentioned in the model description, the four classifiers with the best accuracies were selected to be part of the ensemble module. These four classifiers were the SVM, KNN, LightGBM, and CNN. Figure 3 shows the performance of each of these classifiers with the three embedding methods and confirms that the GPT-3-based embedding outperformed the other methods for all classifiers. Regarding Weighted Voting, Table 3 shows that the classification results of this technique are very interesting and outperformed all other classifiers. The final output of the Weighted Voting reached an Accuracy of 0.999103, a Precision of 1, a Recall of 0.993243, and an F1-score of 0.996610. To give an idea of the distribution of the classification results, we present in Figure 4 the confusion matrix of the four classifiers and the Weighted Voting classification.

### 5.4. Statistical Analysis

To statically verify whether the model proposed in this paper can indeed improve spam message classification, we used the Wilcoxon Signed-Ranks Test technique. This technique is a nonparametric statistical hypothesis test that evaluates the differences in performance of two classifiers for each observation and determines whether the null hypothesis that the performances of the two classifiers are identical can be rejected with a confidence level α [32]. Using this technique, we performed two types of comparisons. The first was to compare the performance of the proposed GPT-3-based text representation technique with the performance of classical representation techniques (TF-IDF and Word embedding) by evaluating the Accuracy of 10 different classifiers. The second was to compare the performance of our proposed Ensemble model with each of the four base learners.

For the first comparison, we considered the null hypothesis $H_{0}$ (the performance using the two embedding techniques are equivalent) and the alternative hypothesis $H_{1}$ (there is a significant difference between the performance of the two techniques). The results of this comparison are presented in Table 4. They show that the Accuracies of the 10 classifiers using the GPT-3 embedding were all significantly superior to those of the classical embedding. Then, two specific values were calculated: *p*-value and z-value. In the Wilcoxon test with a confidence level of 95%, the null hypothesis is rejected if the *p*-value < α = 0.05 and the *z*-value < −1.96. In our case, *p*-value = 0.0019 and *z*-value = −3.097269, which means that the null hypothesis was rejected. Therefore, we can conclude that the results of the GPT-3 embedding technique were statically better than the previous techniques, which confirmed that this embedding technique can significantly improve the classification results.

For the second comparison, we considered the null hypothesis $H_{0}$ (the Ensemble module based on Weighted Voting is equivalent to the base classifier) and the alternative hypothesis $H_{1}$ (there is a significant difference between the two classifiers). We performed four pairwise Wilcoxon tests between the Ensemble with Weighted Voting and each of the four base classifiers (SVM, KNN, LightGBM, and CNN). The goal was to evaluate the difference in Accuracy between the two classifiers through different dataset splits. The *p* and *z* values calculated from these four tests are presented in Table 5. The results show that, for each of the four tests, the *p*-value was less than 0.05, and the *z*-value was less than −1.96. This means that the null hypothesis was rejected and confirmed that the results of classification based on Weighted Voting were statically better than those of the base classifiers. In this way, we can also confirm that the Ensemble method we proposed in this paper can significantly improve the classification results of spam messages.

### 5.5. Discussion

The idea of improving the classification of spam messages that we proposed in the current paper involves two fundamental methods: (i) the use of a recent embedding technique based on the GPT-3 transformer, and (ii) the use of an ensemble strategy with Weighted Voting. As we have already shown in Figure 3, the GPT-3 embedding provided us with a high-quality message representation, which contributed to the improvement of the classification results. By interpreting Table 3 and Figure 4, we can see that the CNN algorithm performed better in terms of Accuracy, Recall, and the F1-score, while the SVM and LightGBM performed better in terms of Precision. This explains that the CNN produced less false negatives, while the SVM and LightGBM producde less false positives. To take advantage of the benefits of each classifier, the application of Ensemble Learning was very useful in this case. This is confirmed in Table 3 by the good results shown from the Weighted Voting classification. This method not only outperformed the classifiers tested in this paper, but it also outperformed the related works. Table 6 compares our results against those described in the related work section.

The convincing results of this model do not preclude some limitations that need to be considered in future works. The dataset tested in this article includes only 5574 English messages. Although it was our best choice compared to the currently available datasets, the model needs to be enriched with more diverse and larger training data. In fact, this model can be easily adapted to languages other than English because of the embedding method used; one just needs to find additional multilingual data and add them to the training phase. On the other hand, the Ensemble Learning method incorporated in the model is somewhat complex. It includes four different classification algorithms; among them is a deep learning classifier. This may affect the performance of the system by increasing the computation time and memory usage. Currently, this issue is not a problem for our model, since SMS are generally short messages that do not require much processing capacity at either the embedding or classification level. However, this observation should be considered if we want to extend the model to support larger messages (e.g., emails) or if we want to use it on devices with limited hardware performance.

## 6. Conclusions

This paper proposed a security solution for SMS spam detection. The model was built on the GPT-3 Transformer and Ensemble Learning. The first objective of the proposed model was to take advantage of the latest NLP Transformers to produce dense and meaningful representations of text messages. The second objective was to apply an Ensemble Learning strategy to create a robust classifier capable of detecting spam messages with high precision. For this task, four classification algorithms were used (SVM, KNN, CNN, and LightGBM), and a Weighted Voting technique was applied to predict the final decision of the Ensemble Learning module. Experimental results showed that our model achieved cutting-edge results with 99.91% Accuracy, 100% Precision, 99.32% Recall, and 99.66% for the F1-score.

We evaluated our solution on English SMS messages, but we believe it can be properly adapted to other environments as well. In the future, we plan to work on the embedding method so that it can support more languages than English. We also plan to extend the training base of the model with other types of messages from social networks such as Twitter and Facebook. In addition, we will work on new solutions to deal with alternative methods of spammers (such as “graphic spam”) that are used to bypass spam detection and filtering systems. 

## Figures and Tables

**Table 1:** A comparative analysis of the discussed related works.

Reference	Main Idea	Used Techniques	Dataset
Ghourabi et al. [9]	SMS classification and spam detection	CNN-LSTM	English SMS spam dataset [15] Private Dataset
Roy et al. [10]	SMS classification and spam detection	CNN-LSTM	English SMS spam dataset [15]
Mishra et al. [6]	Spam detection, content analysis, and URL verification	Naive Bayes	English SMS spam dataset [15] Pinterest Smishing message images
Joo et al. [7]	Content analysis, messages classification, and SMiShing detection	Naive Bayes	private dataset
Sonowal et al. [2]	SMS classification and spam detection	Random Forest, Support Vector Machine, Decision Tree, and AdaBoost	English SMS spam dataset [15] no-English dataset [16]
Almeida et al. [14]	Improve classification results by normalizing and expanding text messages.	Semantic analysis, semantic dictionaries, and disambiguation methods	English SMS spam dataset [15]
Arifin et al. [8]	SMS classification and spam detection	Naive Bayes, FP-growth	English SMS spam dataset [15]
Xia et al. [11]	SMS classification and spam detection	Word weighting and Hidden Markov model	English SMS spam dataset [15] Private Dataset
Ghourabi [12]	SMiShing messages detection	BERT Transformer and MLP network	English SMS spam dataset [15] Private Dataset
Liu et al. [13]	SMS classification and spam detection	Transformer	English SMS spam dataset [15] UtkMl’s Twitter Spam Detection Competition

**Table 2:** Classification results.

Embedding Method	Classifier	Accuracy	Precision	Recall	F1-Score
TF-IDF	SVM	0.978475	0.976923	0.858108	0.913669
	KNN	0.934529	0.987013	0.513514	0.675556
	Decision Tree	0.959641	0.850340	0.844595	0.847458
	Logistic Regression	0.951570	0.919643	0.695946	0.792308
	Random Forest	0.979372	1.000000	0.844595	0.915751
	AdaBoost	0.966816	0.958678	0.783784	0.862454
	Bagging	0.966816	0.899281	0.844595	0.871080
	LightGBM	0.975785	0.911565	0.905405	0.908475
Word Embedding	CNN	0.986547	0.985401	0.912162	0.947368
	LSTM	0.987444	0.971831	0.932432	0.951724
BERT Embedding	SVM	0.993722	0.973154	0.979730	0.976431
	KNN	0.981166	0.894410	0.972973	0.932039
	Decision Tree	0.965919	0.857143	0.891892	0.874172
	Logistic Regression	0.994619	0.973333	0.986486	0.979866
	Random Forest	0.987444	0.985507	0.918919	0.951049
	AdaBoost	0.994619	0.979730	0.979730	0.979730
	Bagging	0.989238	0.985714	0.932432	0.958333
	LightGBM	0.993722	0.986207	0.966216	0.976109
	CNN	0.993722	0.986207	0.966216	0.976109
	LSTM	0.991031	0.979167	0.952703	0.965753
GPT-3 Embedding	SVM	0.996413	1.000000	0.972973	0.986301
	KNN	0.994619	0.973333	0.986486	0.979866
	Decision Tree	0.979372	0.931034	0.912162	0.921502
	Logistic Regression	0.992825	0.986111	0.959459	0.972603
	Random Forest	0.982063	1.000000	0.864865	0.927536
	AdaBoost	0.993722	0.973154	0.979730	0.976431
	Bagging	0.982063	0.950704	0.912162	0.931034
	LightGBM	0.995516	1.000000	0.966216	0.982818
	CNN	0.997309	0.986577	0.993243	0.989899
	LSTM	0.993722	0.993007	0.959459	0.975945

**Table 3:** Comparison of weighted voting ensemble with base classifiers.

Embedding Method	Classifier	Accuracy	Precision	Recall	F1-Score
GPT-3 Embedding	SVM	0.996413	1.000000	0.972973	0.986301
	KNN	0.994619	0.973333	0.986486	0.979866
	LightGBM	0.995516	1.000000	0.966216	0.982818
	CNN	0.997309	0.986577	0.993243	0.989899
	Weighted Voting Ensemble	0.999103	1.000000	0.993243	0.996610

**Table 4:** Accuracy difference between GPT-3 embedding and TF-IDF/Word embedding based on Wilcoxon Signed-Ranks Test. The results yielded *p*-value = 0.0019 and *z*-value = −3.097269.

Classifier	Accuracy		Diff	Rank
	TF-IDF/Word Embedding	GPT-3 Embedding		
SVM	0.978475	0.996413	−0.017938	5
KNN	0.934529	0.994619	−0.06009	10
Decision Tree	0.959641	0.979372	−0.019731	6.5
Logistic Regression	0.95157	0.992825	−0.041255	9
Random Forest	0.979372	0.982063	−0.002691	1
AdaBoost	0.966816	0.993722	−0.026906	8
Bagging	0.966816	0.982063	−0.015247	4
LightGBM	0.975785	0.995516	−0.019731	6.5
CNN	0.986547	0.997309	−0.010762	3
LSTM	0.987444	0.993722	−0.006278	2

**Table 5:** Wilcoxon Signed-Ranks Test results measuring the difference in Accuracy between Ensemble Weighted Voting and base classifiers.

	*z*-Value	*p*-Value
Weighted Voting VS. SVM	−3.030698	0.00243989
Weighted Voting VS. KNN	−3.270020	0.00107540
Weighted Voting VS. LightGBM	−3.021351	0.00251650
Weighted Voting VS. CNN	−2.119996	0.0340064

**Table 6:** Results comparison with previous works.

Reference	Used Techniques	Accuracy
Ghourabi et al. [9]	CNN-LSTM	98.37%
Roy et al. [10]	CNN-LSTM	99.44%
Mishra et al. [6]	Naive Bayes	96.29%
Joo et al. [7]	Naive Bayes	-
Sonowal et al. [2]	Random Forest, Support Vector Machine, Decision Tree, and AdaBoost	96.4%
Almeida et al. [14]	Semantic analysis, semantic dictionaries, and disambiguation methods	-
Arifin et al. [8]	Naive Bayes, FP-growth	98.5%
Xia et al. [11]	Word weighting and Hidden Markov model	96.9%
Ghourabi [12]	BERT Transformer and MLP network	99.45%
Liu et al. [13]	Transformer	98.92%
Our model	Transformer-based embedding and Ensemble Learning	99.91%

## Data Availability

Not applicable.
